# A pan-cancer analysis of the role of argininosuccinate synthase 1 in human tumors

**DOI:** 10.3389/fonc.2023.1049147

**Published:** 2023-11-20

**Authors:** Qiang Ding, Ruiqi Li, Qingming Wang, Li Yu, Fuming Zi

**Affiliations:** ^1^Department of Hematology, The Second Affiliated Hospital of Nanchang University, Nanchang, Jiangxi, China; ^2^Institute of Hematology, Nanchang University, Nanchang, Jiangxi, China; ^3^Key Laboratory of Hematology, The Second Affiliated Hospital of Nanchang University, Nanchang, Jiangxi, China

**Keywords:** pan-cancer analysis, argininosuccinate synthase 1, gene expression analysis, immune infiltration analysis, enrichment analysis of gene function

## Abstract

**Aim:**

There is accumulating evidence indicating that ASS1 is closely related to tumors. No pan-cancer analysis of ASS1 was available.

**Methods:**

Here we explored the gene expression and survival analysis of *ASS1* across thirty-three tumors based on the datasets of the TCGA (Cancer Genome Atlas), the GEO (Gene Expression Omnibus), and the GEPIA2 (Gene Expression Profiling Interactive Analysis, version 2).

**Results:**

*ASS1* is highly expressed in most normal tissues and is related to the progression of some tumors. We also report *ASS1* genetic alteration and their association with tumor prognosis and report differences in ASS1 phosphorylation sites between tumors and control normal tissues. *ASS1* expression was associated with the infiltration of cancer-associated fibroblasts (CAFs) for the TCGA tumors of BRCA (Breast invasive carcinoma), CESC (Cervical squamous cell carcinoma and endocervical adenocarcinoma), COAD (Colon adenocarcinoma), ESCA (Esophageal carcinoma), SKCM (Skin cutaneous melanoma), SKCM-Metastasis, TGCT (Testicular germ cell tumors), and endothelial cell for the tumors of BRCA, BRCA-Basal, CESC, ESCA, KIRC (Kidney renal clear cell carcinoma), LUAD (Lung adenocarcinoma), LUSC (Lung squamous cell carcinoma), SKCM, SKCM-Metastasis, SKCM-Primary, STAD (Stomach adenocarcinoma), and TGCT. The KEGG and GO analysis were used to analyze ASS1-related signaling pathways. Finally, we used Huh7 cell line to verify the function of ASS1 *in vitro*. After ASS1 knockdown using small interfering RNA (siRNA), the proliferation and invasion of Huh7 were enhanced, cyclin D1 was up-regulated, and anti-apoptotic protein bax was down-regulated, suggesting that ASS1 is a tumor suppressor gene in hepatocellular carcinoma.

**Conclusion:**

Our first pan-cancer study offers a relatively comprehensive understanding of the roles of ASS1 in different tumors.

## 1 Introduction

Taking account of the different roles and complexity of the same gene in different tumors, to conduct a pan-cancer analysis of the gene, assess its role in different tumors and its relation to clinical outcomes, as well as the possible molecular mechanisms are necessary. TCGA database and GEO database contain functional genomic datasets of different tumors, which can help us with pan-cancer analysis (1, 2).

One hallmark of cancer is metabolic reprogramming (3). Tumors and stromal cells coexist in the harsh tumor microenvironment (TME). Both tumor and stromal cells undergo rapid metabolic adaptation during tumor progression and metastasis (4, 5). A pivotal requirement for cell growth is the rigorous control of nitrogen acquisition and consumption. Nitrogen can be generated from amino acid catabolism (6). Amino acid metabolism is deregulated in many tumors, with changes in amino acid metabolism affecting the cancer cell state as well as systemic metabolism in individuals with malignancy (7). Arginine is an important amino acid that is central to tumor metabolism (8, 9). Argininosuccinate synthase 1 (ASS1) plays a key role in arginine synthesis and urea cycle which is indispensable for converting excess nitrogen from ammonia and amino acids into urea (6, 10). The silencing of the ASS1 gene that encodes the rate-limiting enzyme in the urea cycle and arginine auxotrophy resulting from the silencing of ASS1 are common metabolic alterations in many cancers (8, 11). Loss of ASS1 expression leads to dependence on extracellular arginine for cell growth, proliferation, and survival, known as arginine auxotrophy (8). Arginine depletion is one of the therapeutic approaches for cancer, which requires the identification of the status of ASS1. ASS1-deficient tumor cells cannot synthesize arginine and may benefit from arginine depletion therapy (12).

In this paper, we used the TCGA database and GEO database to analyze the pan-cancer analysis of ASS1, including the expression of the *ASS1* gene and protein in different tumors, the impact of ASS1 on tumor survival, changes in immune-infiltrating cells and related cellular pathways, etc., to explore the potential molecular mechanism of ASS1 in the pathogenesis or clinical outcomes of different tumors.

## 2 Materials and methods

### 2.1 Gene expression and analysis

We imported the *ASS1* gene into the “Gene_DE” module of the TIMER2 (tumor immune estimation resource, version 2) website (http://timer.cistrome.org/) and observed the expression of the *ASS1* gene in different tumor tissues and related normal tissues in the TCGA database. For tumors without normal control tissues, we used the “Expression analysis-Box Plot” module of the GEPIA2 (Gene Expression Profiling Interactive Analysis, version 2) database (http://gepia2.cancer-pku.cn/#analysis) as a supplement (13) to obtain tumor tissue expression and the corresponding expression in normal tissue of the GTEx (Genotype-Tissue Expression) database, under the settings of *P*-value cutoff = 0.01, log_2_FC (fold change) cutoff =1, and “Match TCGA normal and GTEx data”. Furthermore, we obtained the violin plot of ASS1 expression in different pathological stages (I, II, III, IV) of all tumors with the “Expression analysis-Stage Plot” module of GEPIA2.

We used the UALCAN (http://ualcan.path.uab.edu/analysis-prot.html) website to conduct protein expression analysis with the CPTAC (Clinical proteomic tumor analysis consortium) module (14).

Human Protein Atlas (HPA) is a database that focused on exploring the human protein in cells, tissues, and organs (15). In this study, the protein expression level of breast cancer, renal cancer, colon cancer, GBM (Glioblastoma multiforme), LIHC (Liver hepatocellular carcinoma), ovarian cancer, and UCEC (Uterine corpus endometrial carcinoma) was obtained from the tumor tissues and corresponding normal tissues of the HPA dataset. Multiple myeloma is a tumor of interest to us, and the HPA database lacks the results of myeloma ASS1 immunohistochemical staining (IHC). In order to further explore the protein expression of ASS1 in myeloma bone marrow biopsy specimens, we used IHC staining to detect bone marrow biopsy specimens of newly diagnosed multiple myeloma patients, and the IHC method was referred to previous articles (16). Anti-ASS1 antibody was purchased from Abcam (Abcam, ab170952). Sections were blocked with 10% H_2_O_2_ for 7min and washed with PBS for 3 times. Then incubated with Anti-ASS1 antibody at 1/250 dilution.

### 2.2 Survival prognosis analysis

The “Survival Analysis-Survival Map” module of GEPIA2 was used to obtain the overall survival (OS) and disease-free survival (DFS) data of *ASS1* in all TCGA tumors (13). We used cutoff-high (50%) and cutoff-low (50%) values as the expression thresholds for defining the high-expression and low-expression cohorts. The log-rank test was used for the hypothesis test, and the survival curve was obtained through the “Survival Analysis” module of GEPIA2.

### 2.3 Genetic alteration analysis

Regarding the genetic variation characteristics of the *ASS1* gene, we used the cBioPortal website (https://www.cbioportal.org/) (17) and to select the “TCGA PanCancer Atlas Studies-Query By Gene” in the “Quick select” section and entered “ASS1”. The alteration frequency, mutation type, and CNA (Copy number alteration) of ASS1 in all TCGA tumors were observed in the “Cancer Types Summary” module. The mutated site of ASS1 was displayed in the schematic diagram of the protein structure or the 3D (Three-dimensional) structure via the “Mutations” module. The overall survival, disease-free survival, progression-free survival, and disease-free survival were obtained from the “Comparison” module of the TCGA cancer cases with or without ASS1 genetic alteration. Kaplan-Meier plots with log-rank P-value were generated as well.

### 2.4 Immune infiltration analysis

We used the “Immune-Gene” module of the TIMER2 database to explore the association between ASS1 expression and immune infiltration of all TCGA tumors. The EPIC, MCPCOUNTER, XCELL, TIDE, CIBERSORT, and CIBERSORT-ABS algorithms were applied for immune infiltration estimations. The *P*-values and partial correlation (cor) values were acquired via the purity-adjusted Spearman’s rank correlation test. The data were demonstrated as a heatmap and a scatter plot.

### 2.5 ASS1-related gene enrichment analysis

The STRING website (https://string-db.org/) was used to screen the top 50 interacting proteins that were experimentally validated for binding to ASS1. Enter “ASS1”, select “Homo sapiens” for species, and set the main parameters as follows: Network type: (“Full network”), meaning of network edges (“evidence”), active interaction sources (“Experiments”), the minimum required interaction score [“low confidence (0.150)”], and max number of interactors to show (1st shell: “no more than 50 interactors”). Download the protein data was bound to ASS1 and visualize it with Cytoscape software.

Using the “Expression Analysis-Similar Gene Detection” module of GEPIA2, the top 100 target genes associated with ASS1 were obtained on all TCGA tumor and normal tissue datasets. Simultaneously, the Pearson correlation analysis of coupled genes between ASS1 and the selected genes was performed out using the “Expression Analysis-Correlation Analysis” module of GEPIA2, and the scatter plot was represented by log2 TPM. Additionally, we also used the “Exploration-Gene_Corr” module of TIMER2 to obtain heatmap data for the selected genes.

Intersection analysis was performed using the Jvenn website (http://bioinformatics.psb.ugent.be/webtools/Venn/) to compare the top 100 genes that bind to ASS1 and those that interact with ≤50 genes (18). Then, the two parts of the data were combined for KEGG (Kyoto encyclopedia of genes and genomes) pathway analysis and GO (Gene Ontology) analysis (19). Upload the above list of total genes to the DAVID (Database for Annotation, Visualization, and Integrated Discovery) website (https://david.ncifcrf.gov/) and set the selected condition (“OFFICIAL_GENE_SYMBOL”) and species (“Homo sapiens”). Additionally, we used DAVID’s “Functional Annotation Chart” module to analyze the above gene list, and selected the KEGG pathway and GO analysis, respectively. Download the file and select data with a *p*-value ≤ 0.05. The enriched results were finally visualized on the Bioinformatics website (http://www.bioinformatics.com.cn).

### 2.6 Cell proliferation assays

The Cell Counting Kit-8 (CCK8, Dojindo, Japan) assay was used to assess Huh7 cells and Hep3B cells proliferation. 1×10^3^/well Huh7 cells and Hep3B cells respectively were seeded in 96-well plates and incubated with or without ASS1-siRNA (Sangon, Shanghai, China; hASS1-1545, sense(5′→3′): CUCAGGCUGAAGGAAUAUCAUTT, antisense(5′→3′): AUGAUAUUCCUUCAGCCUGAGTT) with lipofectamine™ RNAiMAX (Invitrogen, USA) at 37°C in a humidified atmosphere and 5% CO_2_. After incubation for 24, 48, and 72 hours, cells were treated with 10 μL CCK8 solution and incubated at 37°C for another 2 hours. Then, absorbance was measured at 450 nm using a microplate reader (PerkinElmer, USA).

BrdU Flow Kits (BD Pharmingen™, America) assay was used to assess Huh7 cells and Hep3B cells proliferation. (1).Resuspend the cells in 100 µL of BD Cytofix/Cytoperm Buffer per tube. Incubate the cells for 15 to 30 minutes on ice. Wash the cells with 1 mL of 1 × BD Perm/Wash Buffer. Centrifuge for 5 minutes at 200 to 300g and discard the supernatant. (2). Resuspend the cells in 100 µL of BD Cytoperm Permeabilization Buffer Plus per tube. Incubate the cells for 10 minutes on ice. Wash the cells in 1 mL of 1 × BD Perm/Wash Buffer. (3) Resuspend the cells in 100 µL of BD Cytofix/Cytoperm Buffer per tube. Incubate the cells for 5 minutes at on ice. Wash the in 1 mL of 1 × BD Perm/Wash Buffer. (4). Resuspend the cells in 100 µL of diluted DNase (diluted to 300 µg/mL in DPBS) per tube. Incubate cells for 1 hour at 37°C. Wash the cells in 1 mL of 1 × BD Perm/Wash Buffer. (5). Resuspend the cells in 50 µL of BD Perm/Wash Buffer containing diluted fluorescent anti-BrdU. Incubate the cells for 20 minutes at room temperature. Wash the cells in 1 mL of 1 × BD Perm/Wash Buffer.

### 2.7 Wound healing assays

2×10^6^ Huh7 cells and Hep3B cells respectively were seeded in the 6-well plate. After 24-48h, the cells grew to about 90%-100% confluence. And then, the cell layer was scraped straightly with pipette tips. Twice photographing was performed at a same region of the scratched line of the cell after 0h and 24h (Leica Imaging system, Germany).

### 2.8 Migration assay

Huh7 cells and Hep3B cells migration was performed as previously described (20). Briefly, Huh7 cells and Hep3B cells were transfected ASS1-targeted siRNA when the confluency of the cells reached about 70% in 6-cm dishes. Six hours later, the culture medium was replaced with fresh RPMI1640 medium, and cultured for another 24h. Then, 5x10^5^/ml of Huh7 cells and Hep3B cells were digested and 100ul suspension was loaded in the upper chamber (3%FBS) of the transwell (lower chamber, 20%FBS) (8-µm pore; Becton Dickinson, USA). The plates were then cultured at 37˚C in 5% CO2 for 48 h. The cells migrated across the membrane and adhered to the lower part of the membrane, while those that did not migrate were removed with a cotton swab. The former were stained with crystal violet (0.1%) and examined with microscopy.

### 2.9 Western blotting

The western blotting was performed as described previously (16). Briefly, 48h after transfection with hASS1-siRNA, Huh7 cells and Hep3B cells protein were collected with the lysis buffer (RIPA, Beyotime). All samples were analyzed with SDS-PAGE gel (Genescript, China). After blocking with 3% BSA, membranes were incubated with the corresponding primary antibodies overnight at 4°C. After incubation with antibodies, the bands of the proteins were stained with ECL reagent (Millipore, US) and were captured with AI600 imager (GE, US). The antibodies, cyclin D1, BAX, ASS1 and GAPDH, were purchased from Proteintech Group, Inc.(Chicago, USA).

## 3 Results

### 3.1 Gene expression analysis data

**Figure 1:**
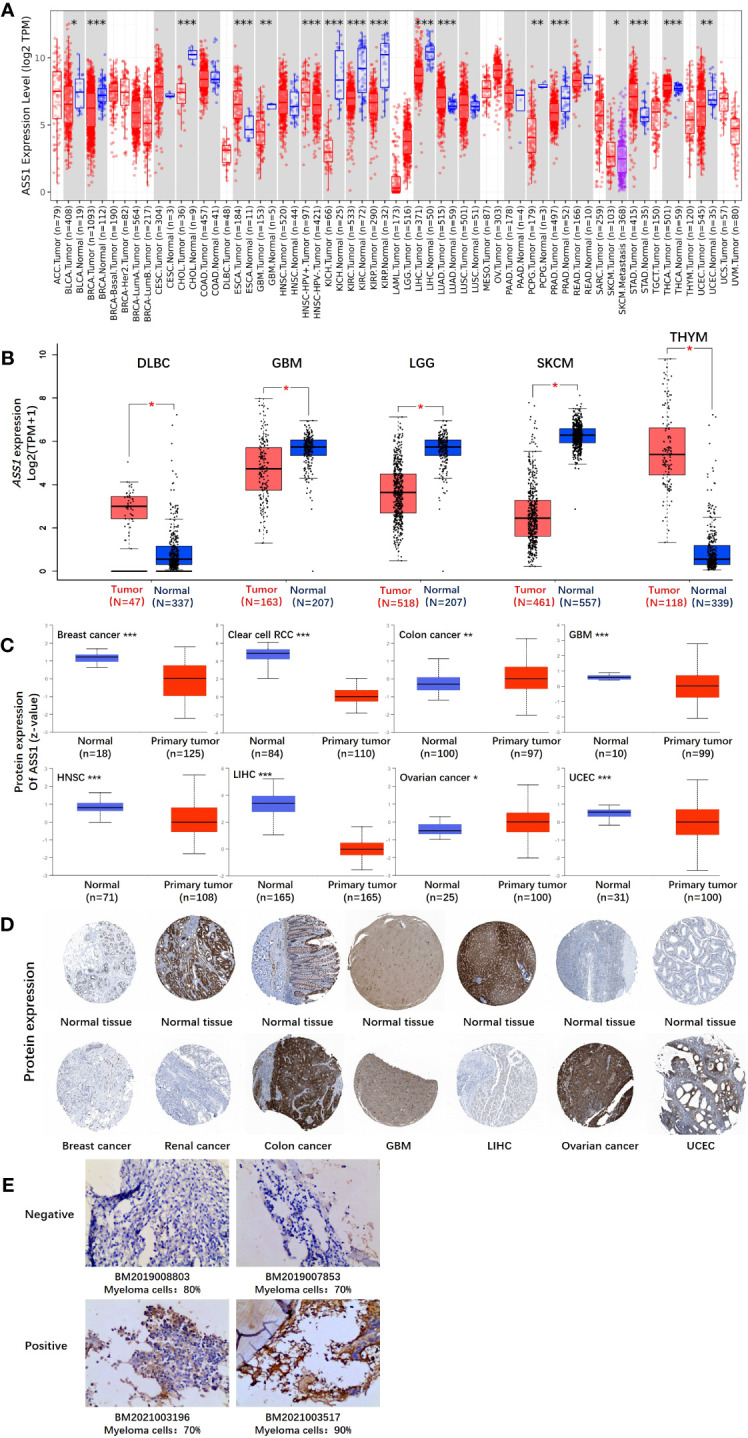
The expression level of the *ASS1* gene in different tumors and pathological stages. **(A)** The expression status of the *ASS1* gene in different tumors or specific tumor subtypes was analyzed through TIMER2. *P<0.05; **P<0.01; ***P<0.001. **(B)** For the types of DLBC, GBM, LGG, SKCM, and THYM in the TCGA project, the corresponding normal tissues in the GTEx database were included as controls. The box plot data were supplied. **P<0.01. **(C)** The expression level of ASS1 total protein based on the CPTAC dataset between normal tissue and primary tissue of breast cancer, clear cell RCC, colon cancer, GBM, HNSC, LIHC, ovarian cancer, and UCEC. ***P<0.001. **(D)** The expression level of ASS1 total protein is based on the HPA dataset. **(E)** ASS1 expression in multiple myeloma bone marrow biopsy specimens.

In this study, we explored the role of human ASS1 (NM_000050 for mRNA or NP_000041 for protein) in tumors. We first analyzed the expression of ASS1 in different tumors and non-tumor tissues. The TIMER2 database was used to analyze the expression of the *ASS1* gene in various TCGA tumors. As shown in **Figure 1A**, the expression of *ASS1* in the tumor tissues of ESCA, LUAD, STAD, and THCA (Thyroid carcinoma) (*p*<0.001 for the above tumors) is higher than the corresponding normal tissues. While, the expression of *ASS1* in the tumor tissues of BRCA, CHOL (Cholangiocarcinoma), KICH (Kidney Chromophobe), KIRC, KIRP (Kidney renal papillary cell carcinoma), LIHC, PRAD (Prostate adenocarcinoma) (*p*<0.001 for the above tumors), GBM, PCPG (Pheochromocytoma and paraganglioma), UCEC (*p*<0.01 for the above tumors), and BLCA (Bladder urothelial carcinoma) (*p*<0.05) is lower than the corresponding normal tissues.

Using the GTEx database as a supplement to the TIMER2 database, we further evaluated the expression of *ASS1* in ACC (Adrenocortical carcinoma), DLBC (Diffuse large B-cell lymphoma), GBM, LGG (Brain lower grade glioma), SKCM, TGCT, THYM (Thymoma) and UCS (Uterine carcinosarcoma) between normal tissue and tumor tissue. As shown in **Figure 1B**, the expression of *ASS1* in DLBC and THYM is higher than in the corresponding normal tissues, while that in GBM, LGG and SKCM is lower than in the corresponding normal tissues (*p*<0.01).

The results of the CPTAC datasets showed higher expression of ASS1 total protein in the primary tissue of breast cancer, clear cell RCC, GBM, HNSC (Head and neck squamous cell carcinoma), LIHC, and UCEC (**Figure 1C**, *p*<0.001) than in normal tissues. We further verified the above results by the HPA database as demonstrated in **Figure 1D**. The “Pathological Stage Plot” module of GEPIA2 was used to analyze the correlation between *ASS1* expression and the pathological stages of tumors. The results showed that there was no statistical difference (Data not displayed). As demonstrated in **Figure 1E**, ASS1 expression in bone marrow biopsy specimens of newly diagnosed multiple myeloma patients is different. Some patients have positive ASS1 while others have negative ASS1, indicating that ASS1 expression should be clearly defined before arginine deprivation therapy is used, rather than making a blanket decision on whether arginine deprivation therapy is used or not. We hold the opinion that in multiple myeloma, the use of ADI-PEG20 should be individualized based on ASS1 expression.

### 3.2 Survival analysis data

**Figure 2:**
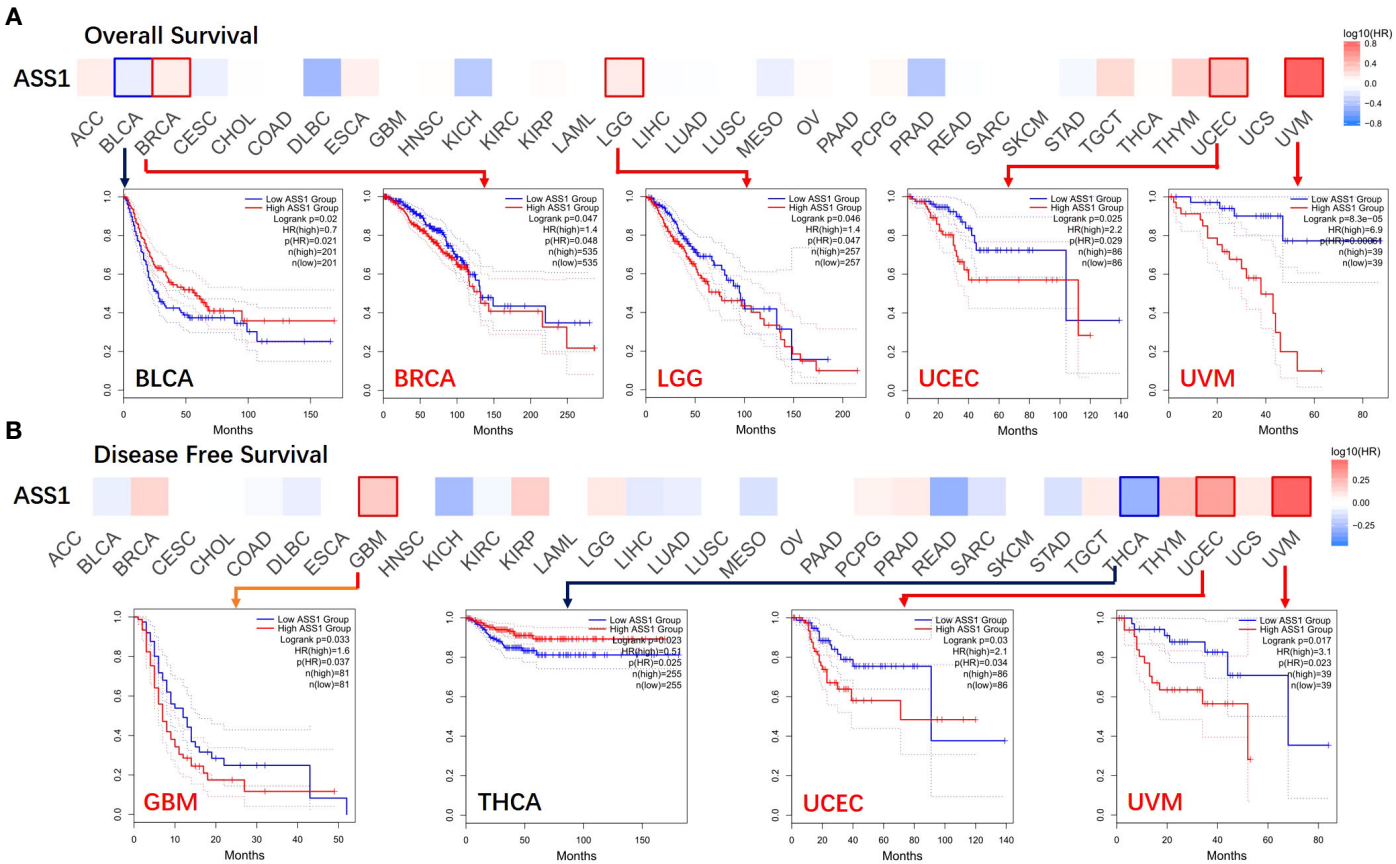
Correlation between *ASS1* gene expression and survival prognosis of cancers in TCGA. The GEPIA2 was used to perform overall survival **(A)** and disease-free survival **(B)** analyses of different tumors in TCGA by *ASS1* gene expression. The survival map and Kaplan-Meier curves with positive results are given.

According to the expression levels of *ASS1*, we divided the cancer cases into high-expression and lower-expression groups, and used TCGA and GEO datasets to study the correlation between *ASS1* expression and the prognosis of different tumor patients. As shown in **Figure 2**, the expression of *ASS1* in most TCGA tumors did not affect OS and DFS. But high expression of *ASS1* in BRCA, LGG, UCEC, and UVM (Uveal melanoma) patients was associated with poor OS (*p*=0.048, *p*=0.047, *p*=0.029, *p*=0.00064, respectively). Only in BLCA patients, a high expression of *ASS1* was associated with better OS (*p*=0.021). In terms of DFS, high expression of *ASS1* in GBM, UCEC, and UVM patients was associated with poor DFS (*p*=0.037, *p*=0.034, *p*=0.023, respectively). While in THCA patients, high expression of *ASS1* was associated with better DFS (*p*=0.025). These results suggest that *ASS1* expression level has different effects on survival in different tumors.

### 3.3 Genetic alteration analysis data

**Figure 3:**
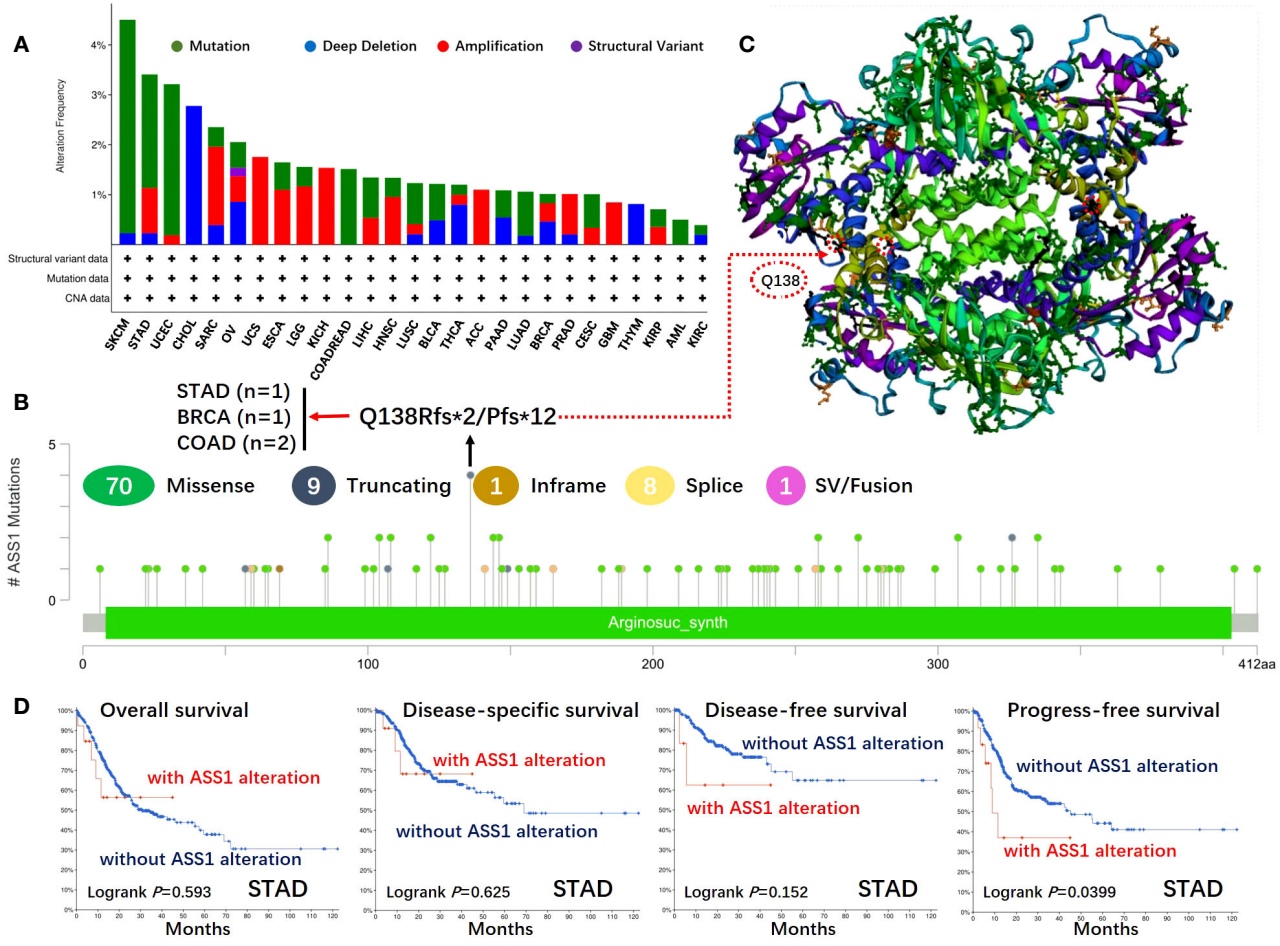
Mutation features of ASS1 in different tumors of TCGA. The mutation features of ASS1 for the TCGA tumors were analyzed using the cBioPortal tool. The alteration frequencies with mutation type **(A)** and mutation site **(B)** are displayed. The mutation site (Q138) in the 3D structure of ASS1 is also displayed **(C)**. The potential correlation between mutation status and overall, disease-specific, disease-free, and progression-free survival of STAD **(D)** was also analyzed using the cBioPortal tool.

The gene changes of *ASS1* in different tumor samples of the TCGA were analyzed. As shown in **Figure 3A**, the highest alteration frequency of *ASS1* (~4%) appears for patients with SKCM and UCEC with “mutation” as the primary type. The “deep deletion” type of CNA was the primary type in the CHOL, which showed an alteration frequency of ~3%. The “amplification” type of CNA was the primary type in the UCS, KICH, ACC, and GBM, which showed an alteration frequency of >1%. The types, locus, and the number of cases of *ASS1* genetic alteration are further illustrated in **Figure 3B**. As shown in **Figure 3B**, the missense mutation of *ASS1* was the main type of genetic alteration. We can also observe that Q138Rfs*2/Pfs*12 alteration was detected in cases of STAD, BRAC (Breast invasive carcinoma), and COAD, which could induce a frameshift mutation of the *ASS1* gene, translation from Q (Glutarnine) to R (Arginine) or P (Proline) at the 138 locus of ASS1 protein. The 3D structure of ASS1 protein is demonstrated in **Figure 3C** of the Q138 site. Additionally, we also explored the relationship between *ASS1* gene alteration and the survival prognosis in different types of cancer. The data in **Figure 3D** indicate that compared with patients without ASS1 alteration, STAD patients with ASS1 alterations had poor progression-free survival (PFS) (*p*=0.0399), while there were no differences in OS (*p*=0.593), disease-specific survival (DSS) (*p*=0.625), DFS (*p*=0.152).

### 3.4 Protein phosphorylation analysis data

**Figure 4:**
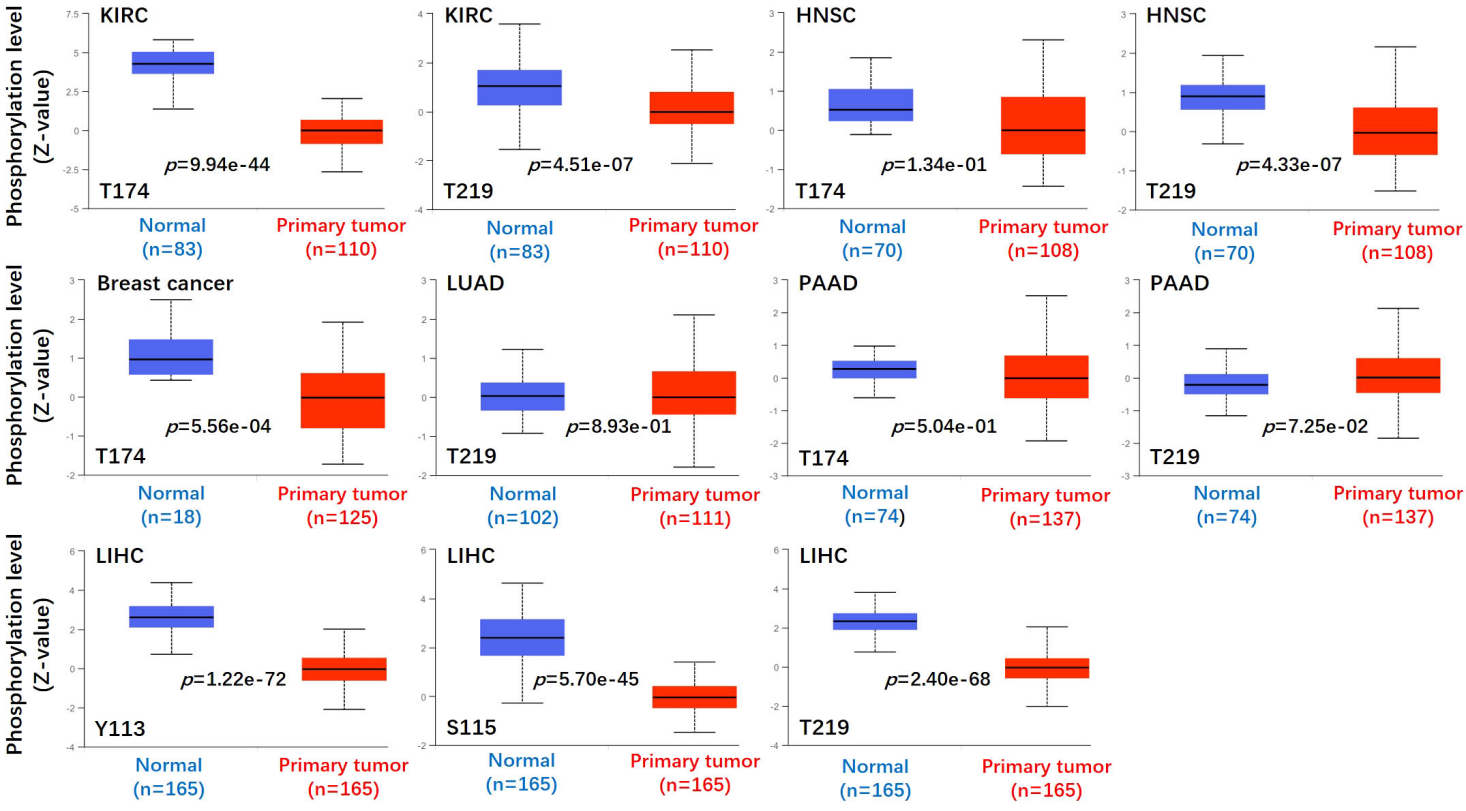
Phosphorylation analyses of ASS1 protein in different tumors. Based on the CPTAC dataset, we analyzed the expression level of ASS1 phosphoprotein (NP_000041, T174, T219, Y113, and S115 sites) between normal tissue and primary tissue of selected tumors via the UALCAN.

We used the CPTAC module of the UALCAN website (http://ualcan.path.uab.edu/analysis-prot.html) to identify the protein phosphorylation sites and expression levels of ASS1 in tumors. As shown in **Figure 4**, the phosphorylation sites of ASS1 in tumors mainly involved T174, T219, Y113, and S115. For KIRC, HNSC, and PAAD (Pancreatic adenocarcinoma), phosphorylation sites mainly involved T174 and T219. For breast cancer and LUAD, phosphorylation sites mainly involved T314 and T219, respectively. For LIHC, phosphorylation sites mainly involved Y113, S115, and T219.

### 3.5 Immune infiltration analysis data

**Figure 5:**
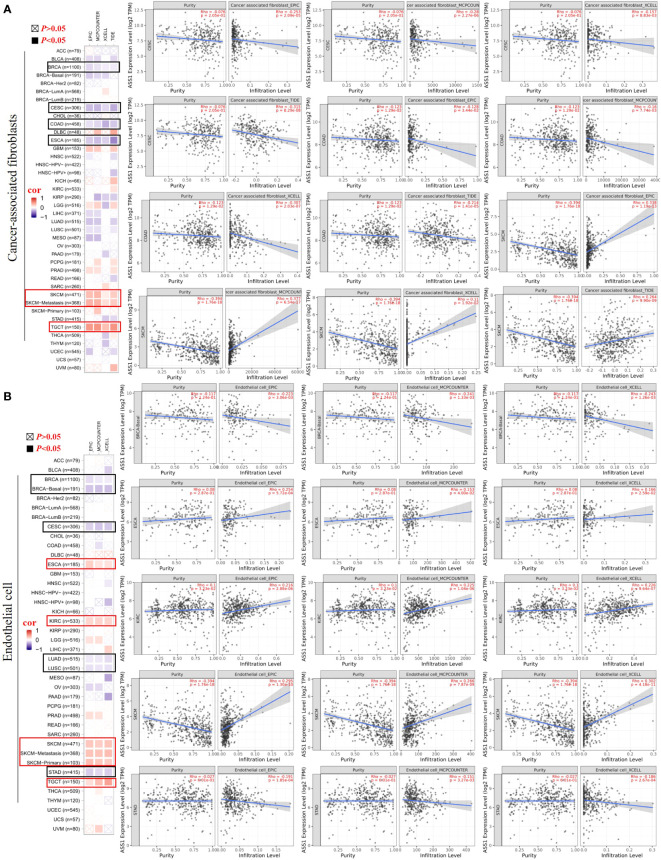
Correlation analyses between ASS1 expression and immune in filtration of cancer-associated fi broblasts **(A)** and endothelial cells **(B)**. Different algorithms were used to explore the potential correlation between the expression level of the ASS1 gene and the in filtration level of cancer-associated fibroblasts and endothelial cells across all types of cancer to TCGA.

It is trusted that the “tumor microenvironment is not just a silent bystander, but rather an active promoter of cancer progression” (21). Tumor-infiltrating immune cells such as T cells, B cells, neutrophils, cancer-associated fibroblasts (CAFs), tumor-associated macrophages (TAMs), dendritic cells (DCs), myeloid-derived suppressor cells (MDSCs) and endothelial cells are important components of the tumor microenvironment (22–24). Therefore, we used the TIMER, CIBERSORT, CIBERSORT-ABS, TIDE, XCELL, MCPCOUNTER, and EPIC algorithms to explore the potential relationship between the infiltration of immune cells and *ASS1* gene expression in different types of TCGA tumors. As demonstrated in **Figure 5A**, we found a positive correlation between ASS1 expression and the infiltration of CAFs for tumors of SKCM, SKCM-metastasis, TGCT, and a negative correlation between BRCA, CESC, COAD, and ESCA. Endothelial cells are another important tumor-infiltrating immune cells in the tumor microenvironment (25–27). As demonstrated in **Figure 5B**, we observed a positive correlation between ASS1 expression and the infiltration of endothelial cells for tumors of ESCA, KIRC, SKCM, SKCM-metastasis, SKCM-primary, TGCT and a negative correlation of BRCA, BRCA-Basal, CESC, LUAD, LUSC, STAD.

### 3.6 Enrichment analysis of ASS1-related partners

**Figure 6:**
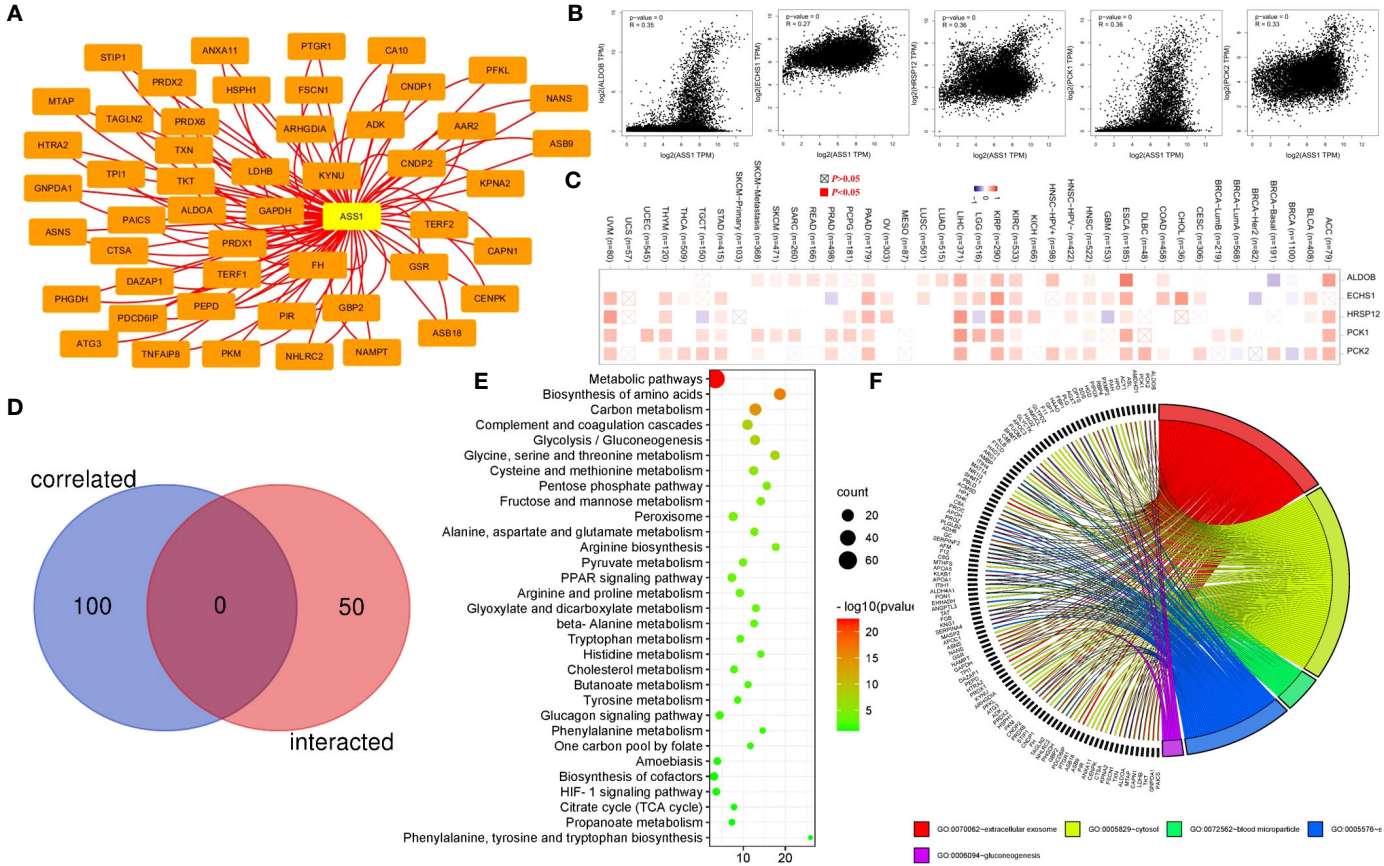
ASS1-related gene enrichment analysis. **(A)** The STRING database was used to obtain the available experimentally verified ASS1-binding proteins. **(B)** Using the GEPIA2 database, we obtained the top 100 ASS1-correlated genes in TCGA projects and analyzed the expression correlation between ASS1 and selected targeting genes, including ALDOB, ECHS1, HRSP12, PCK1, and PCK2. **(C)** The corresponding heatmap data in the detailed cancer types are displayed. **(D)** An intersection analysis of the ASS1-binding and correlated genes were conducted. **(E)** Based on the ASS1-binding and interacted genes, KEGG pathway analysis was performed. **(F)** Based on the ASS1-binding and interacting genes, GO analysis was performed.

To reveal the mechanism of the *ASS1* gene in tumorigenesis, we screened the top 50 proteins interacting with ASS1 and the top 100 genes interacting with ASS1 for enrichment analysis. Using the STRING database, we obtained 50 ASS1-binding proteins, and the interaction network of these proteins is shown in **Figure 6A**. Further, we combined all tumor expression data from TCGA using the GEPIA2 tool to obtain the top 100 genes interacting with ASS1. As shown in **Figure 6B**, the top five genes positively correlated with ASS1 expression are ALDOB (Aldolase B) (R=0.35), ECHS1 (Enoyl Coenzyme A hydratase, short chain, 1) (R=0.27), HRSP12 (Heat-responsive protein 12) (R=0.36), PCK1 (Phosphoenolpyruvate carboxykinase 1) (R=0.36) and PCK2 (Phosphoenolpyruvate carboxykinase 2) (R=0.33) genes (all *p <*0.001). The heatmap data are shown in **Figure 6C**, demonstrating a positive correlation between ASS1 and the above five genes in most cancer types. The cross-analysis of the two groups revealed no common member. (**Figure 6D**).

To further study the signaling pathways involved in ASS1 and its related genes, we combined the two datasets for KEGG enrichment analysis. The results are shown in **Figure 6E**. The above genes are mainly enriched in metabolic pathways, biosynthesis of amino acids, carbon metabolism, complement and coagulation cascades, and glycolysis/gluconeogenesis. Simultaneously, we performed GO analysis on the above genes, and according to the *p*-value, the top 5 GO terms: GO:0070062~extracellular exosome, GO:0005829~cytosol, GO:0072562~blood microparticle, GO:0005576~extracellular region, GO:0006094~gluconeogenesis were mapped, and the results are shown in **Figure 6F**.

### 3.7 Functions of ASS1 *in vitro*

**Figure 7:**
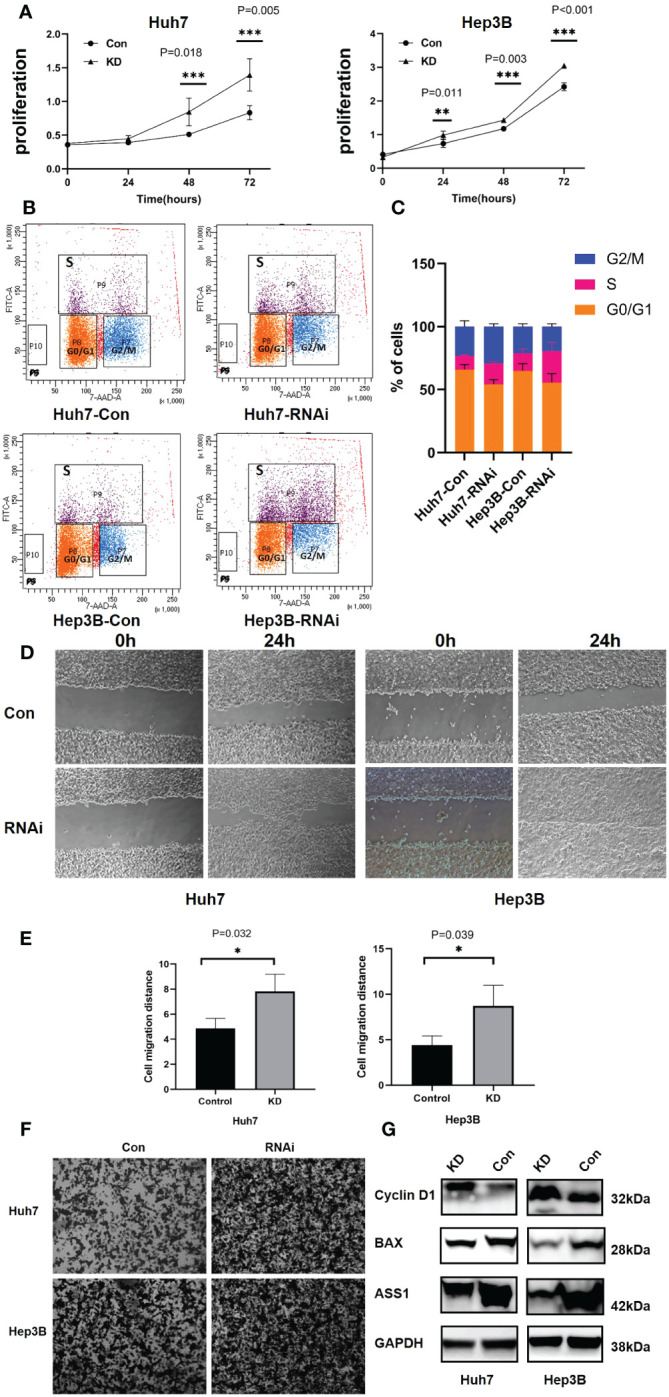
Functions of ASS1 *in vitro.* **(A)** Cell proliferation in Huh7 and Hep3B cell lines at 24, 48, and 72 hours after treatment with ASS1-siRNA (hASS1-1545) detected by CCK8. The data were obtained from three independent experiments and are presented as mean ± SD. **(B)** The BrdU assay revealed that the proportion of S-phase cells increased after ASS-siRNA was added to Huh7 and Hep3B cells. **(C)** The statistical results of Brdu assay. **(D)** The wound healing assays of Huh7 and Hep3B cell lines with or without ASS1-siRNA (hASS1-1545) at 0h and 24h respectively. The data were obtained from three independent experiments and representative results are presented. **(E)** The statistical results of wound healing. **(F)** Transwell assays of Huh7 and Hep3B cell lines with or without ASS1-siRNA (hASS1-1545) after 48h respectively. The data were obtained from three independent experiments and representative results are presented. **(G)** Western blot showed that cyclin D1 was up-regulated and bax was down-regulated after knockdown of ASS1. KD: hASS1-1545: sense (5’-3’): CUCAGGCUGAAGGAAUAUCAUTT; antisense (5’-3’): AUGAUAUUCCUUCAGCCUGAGTT.

To detect the function of ASS1, ASS1-siRNA was used to detect the proliferation, invasion, its effect on cyclin D1 and bax of hepatocellular carcinoma cell line Huh7 and Hep3B. As shown in **Figure 7**, after ASS1 knockdown, the proliferation, migration, and invasion ability of Huh7 and Hep3B cells were enhanced. Western blot results showed that cyclin D1 was up-regulated and bax was down-regulated after knockdown of ASS1.

Brdu assay results revealed that after ASS1 knockout, S phase cells of Huh7 and Hep3B increased significantly, suggesting that cyclin D1 promoted G1 phase cells to enter S phase, which was consistent with the results of western blot.

## 4 Discussion

Many anti-tumor therapies have been developed based on differences in tumor cell metabolism (7, 28). The arginine metabolism pathway is central to regulating various tumor metabolism and several molecules involved in this pathway are potential targets for chemoprophylaxis and targeted tumor therapy (7, 12). ASS1 is the main enzyme of arginine metabolism (29). However, the regulatory role of arginine in various cancers remains controversial (12). Previous studies on the role of arginine in cancer treatment have given conflicting results. Some studies have confirmed that arginine can promote tumor growth, but others suggest that arginine is a suitable candidate for cancer treatment (8, 30). Similarly, the role of ASS1 in tumors is also controversial, with some studies showing that ASS1 downregulation has been shown to support cell proliferation, while others showing that ASS1 expression is associated with poor prognosis in patients (10). Whether ASS1 plays a role in different tumors through common molecular mechanisms is unclear. Accordingly, it is essential to perform a global analysis of ASS1 in tumors to understand the metabolic pathways and enzymes involved in the synthesis of arginine metabolites when it comes to cancer therapy. Therefore, we examined ASS1 genes in a total of 33 different tumors based on TCGA, CPTAC, and GEO database data, as well as molecular characteristics of gene expression, survival prognosis, genetic alteration, and immune infiltration.

For lung cancer, ASS1 is overexpressed in LUAD as compared to corresponding normal tissue. However, our database analysis showed that this differential expression did not affect the OS and DFS of LUAD patients. Previous studies have shown that lung cancer with high ASS1 expression is associated with poor survival (10). Giatromanolaki et al. revealed that ASS1 was expressed mainly by cancer cells of non-small-cell lung cancer (NSCLC) (75/98 cases; 76.5%) and patients with ASS1 expression by cancer cells had a better prognosis (31). *In vitro* experiments revealed that glucose deprivation led to a c-MYC-dependent increase in ASS1 (10). Another study showed that approximately 45% of small cell lung cancers and 50% of cell lines assessed were negative for ASS1 and ASS1-deficient small cell lung cancers were sensitive to ADI-PEG20 (pegylated arginine deiminase) (32). Locke et al. generated a model of ADI-PEG20 resistance through the re-expression of ASS1 via demethylation of the ASS1 promoter in mesothelioma cells (33). They showed that ASS1-deficient cells have decreased levels of acetylated polyamine metabolites, with a compensatory increase in the expression of polyamine biosynthetic enzymes (33). In conclusion, the expression of ASS1 in lung cancer and its relationship with prognosis still needs to be further explored.

For breast cancer, our data demonstrated that the expression of ASS1 in the tumor tissues of BRCA is lower than the corresponding normal tissues, and high ASS1 expression is associated with poor overall survival. *In vitro* studies revealed that glucose deprivation or hypoxic conditions led to an ASS1 RNA expression that significantly upregulated and developed ADI resistance (10, 34). Qiu et al. reported that ASS1 was either low in abundance or absent in more than 60% of 149 random breast cancer bio-samples and low ASS1 abundance is prognostic of poor breast cancer survival (35). They found that prolonged arginine starvation by exposure to ADI-PEG20 induced autophagy-dependent death of ASS1-deficient breast cancer cells along with a decrease in mitochondrial oxidative phosphorylation (35). Keshet et al. discovered that high ASS1-expressing breast cancer mice show significantly lower objective response rates to anti-PD-1 therapy and patients with breast cancer with high ASS1 levels have more metastases (10). However, other papers have reported that ASS1 decreased progressively with tumorigenesis and metastasis (36).

For hepatocellular carcinoma, according to the bioinformatics analysis, we and other researchers have found that the expression of ASS1 in LIHC is lower than the corresponding normal tissues (10, 37). The expression of ASS1 isn’t associated with the overall OS and DFS of LIHC, as shown in **Figure 2**. As an arginine auxotrophy tumor, LIHC is a suitable candidate for arginine deprivation therapy. A phase III randomized study showed that ADI-PEG20 monotherapy did not demonstrate an OS benefit in a second-line setting for hepatocellular carcinoma, but demonstrated a trend of improved OS for those with more prolonged arginine depletion (38). Kim et al. showed that high levels of ASS1 were associated with favorable OS in LIHC patients (39). ASS1 overexpression effectively inhibited tumor growth and strengthened the efficacy of combination chemotherapy via activation of the PERK/eIF2α/ATF4/CHOP axis, which is independent of the status of p53 and arginine metabolism (39). There are also contrary conclusions to the above studies. One study showed that down-regulation of ASS1 due to DNA methylation was correlated with poor prognosis in hepatocellular carcinoma patients. Stable silencing of ASS1 promoted the migration and invasion of hepatocellular carcinoma cells (40). Another study demonstrated that ASS1 silencing in hepatocellular carcinoma cell lines is associated with simultaneous cisplatin resistance (41). Our results demonstrated that ASS1 knockdown using siRNA promote the proliferation and invasion of Huh7, cyclin D1 was up-regulated, and bax was down-regulated, suggesting that ASS1 is a tumor suppressor gene in hepatocellular carcinoma.

Glioblastoma is another disease in which arginine metabolism is well studied. Our study found that the expression of ASS1 in GBM is deficient and high ASS1 expression is associated with poor DFS. Previous research demonstrated that GBM patients with ASS1 methylation had downregulation of *ASS1* mRNA and sensitized cells to autophagy upon arginine deprivation (42).

For hematology malignant diseases, methylation in the CpG island of ASS1 has been detected in 71.7% of multiple myeloma patients and patients with ASS1 methylation were less likely to have bone disease and extramedullary disease (43). Arginine starvation induces fast and highly efficient cell death in arginine-auxotrophic myeloma cells (44). In leukemia, the acute myeloid leukemia cells (AMLs) lack ASS1 expression, and deprivation of arginine by ADI-PEG20 reduces AML tumor burden *in vivo* and *in vitro (* 45). Previous work by Gong et al. demonstrated that arginine deiminase is about 100-fold more potent than L-asparaginase in inhibiting the proliferation of lymphatic leukemia cell lines (46). In lymphoid malignancies, hypermethylation of the ASS1 promoter has been observed and led to sensitivity to arginine deiminase treatment with ADI-PEG20 (47).

In this research, we reported the relationship between *ASS1* gene alteration and the survival prognosis of different tumor types. However, there was no statistically significant difference in the survival between ASS1 mutations and various tumors as demonstrated in **Figure 3**.

The composition of the tumor microenvironment varies by tumor type, but the main features include immune cells, stromal cells, blood vessels, and extracellular matrix (24). Understanding the interaction of immune cells and ASS1-expressing tumor cells in the microenvironment is important for predicting immunotherapeutic efficacy and potential resistance mechanism (6). Therefore, we adopted multiple immune deconvolution methods to explore the potential relationship between the infiltration of immune cells and ASS1 gene expression in different types of TCGA tumors. As demonstrated in **Figures 5A, B**, we found a positive correlation of ASS1 expression of SKCM, SKCM-metastasis, and TGCT for infiltrating CAFs and ESCA, KIRC, SKCM, SKCM-metastasis, SKCM-primary, TGCT for infiltrating endothelial cells, and a negative correlation of BRCA, CESC, COAD, ESCA for CAFs and BRCA, BRCA-Basal, CESC, LUAD, LUSC, STAD for endothelial cells. We can also use other methods to analyze tumor immunoinfiltration as mentioned in this paper (48). As Keshet *at al.* show that cells with high ASS1 exhibit reduced immunogenicity through the decrease in expression of low-molecular mass protein-7 (LMP-7) (10). They also revealed that treating patients with high-ASS1 tumors with purine synthesis inhibition is beneficial to immune checkpoint inhibition therapy (10).

In this study, we presented ASS1-binding proteins and ASS1 expression-related genes across all tumors for a series of enrichment analyses and revealed the potential impact of “Metabolic pathways”, “Biosynthesis of amino acids” and “Carbon metabolism” in the pathogenesis of cancers.

In conclusion, our first pan-cancer analysis of ASS1 showed that the expression of ASS1 in most tumors was different from that in normal tissues, and was statistically correlated with clinical prognosis and immune cell infiltration across multiple tumors. Before using ADI-PEG20, we should first determine whether it is an ASS1-deficient tumor or not. The above results can help us understand the role of ASS1 in tumorigenesis from the perspective of clinical tumor samples.

## Data availability statement

All data generated or analyzed during this study are available in the following databases:(http://timer.cistrome.org/, http://gepia2.cancer-pku.cn/#analysis, http://ualcan.path.uab.edu/analysis-prot.html, https://www.proteinatlas.org/humanproteome/pathology, https://www.cbioportal.org/, https://string-db.org/, http://bioinformatics.psb.ugent.be/webtools/Venn/, https://david.ncifcrf.gov/, http://www.bioinformatics.com.cn).

## Ethics statement

Ethical approval was not required for the studies on humans in accordance with the local legislation and institutional requirements because only commercially available established cell lines were used.

## Author contributions

FZ and QD planned the research. FZ and RL performed most datasets. He received help from LY and QW. FZ and QD wrote the manuscript, with significant contributions and edits from LY and QW. All authors contributed to the article and approved the submitted version.
